# Transgenic *Arabidopsis thaliana* containing increased levels of ATP and sucrose is more susceptible to *Pseudomonas syringae*

**DOI:** 10.1371/journal.pone.0171040

**Published:** 2017-02-02

**Authors:** Renshan Zhang, Hua Qi, Yuzhe Sun, Shi Xiao, Boon Leong Lim

**Affiliations:** 1 School of Biological Sciences, the University of Hong Kong, Pokfulam, Hong Kong, China; 2 State Key Laboratory of Biocontrol and Guangdong Key Laboratory of Plant Resources, School of Life Sciences, Sun Yat-sen University, Guangzhou, China; 3 State Key Laboratory of Agrobiotechnology, The Chinese University of Hong Kong, Shatin, Hong Kong, China; Hainan University, CHINA

## Abstract

Disease resistance exerts a fitness cost on plants, presumably due to the extra consumption of energy and carbon. In this study, we examined whether transgenic *Arabidopsis thaliana* with increased levels of ATP and sucrose is more resistant or susceptible to pathogen infection. Lines of *A*. *thaliana* over-expressing purple acid phosphatase 2 (AtPAP2) (OE lines) contain increased levels of ATP and sucrose, with improved growth rate and seed production. Compared to wild type (WT) and *pap2* lines, the OE lines were more susceptible to several *Pseudomonas syringae* pv. *tomato (Pst)* strains carrying *AvrRpm1*, *AvrRpt2 AvrRps4*, *AvrPtoB*, *HrcC* and WT strain DC3000. The increased susceptibility of the OE lines to *Pst* strains cannot solely be attributed to the suppressed expression of R-genes but must also be attributed to the suppression of downstream signaling components, such as *MOS2*, *EDS1* and *EDS5*. Before infection, the levels of salicylic acid (SA) and jasmonic acid (JA) precursor OPDA were similar in the leaves of OE, *pap2* and WT plants, whereas the levels of JA and its derivative JA-Ile were significantly lower in the leaves of OE lines and higher in the *pap2* line. The expression of JA marker defense gene *PDF1*.*2* was up-regulated in the OE lines compared to the WT prior to *Pst* DC3000 infection, but its expression was lower in the OE lines after infection. In summary, high fitness *Arabidopsis thaliana* exhibited altered JA metabolism and broad suppression of R-genes and downstream genes as well as a higher susceptibility to *Pst* infections.

## Introduction

The success of plants in growth and producing offspring is one of the criteria of plant fitness [1]. Plants are continuously exposed to various types of biotic and abiotic stresses. To cope with these stresses, plants invest energy in various stress-related cellular and biochemical processes. There is a continuous competition for resources between growth and reproduction on one hand and defense-related processes on the other [2]. The reduction in growth and yield to cope with various types of stresses is defined as the fitness cost [3]. In a pioneering field experiment with *Arabidopsis thaliana*, it was shown that to protect plants from potential microbial infection by maintaining a resistance (*R*) gene, *RPM1*, in the genome, the *RPM1*^+^ lines exhibited lower shoot biomass and 9% reduction in seed yield at maturity than the *RPM1*^-^ isogenic lines, even in the absence of infection [3]. It was hypothesized that passive and active defense deprived resources (energy and carbon) from growth and development. Sucrose has been regarded as a signaling molecule in plant defense [4]. Exogenous sucrose application was shown to induced PR2 and PR5 mRNA expression in *Arabidopsis thaliana* through a SA-dependent but NPR1-independent signaling pathway [5]. The hypotonic feeding of rice seedlings with sucrose was shown to enhance PR gene expression, and the seedlings sprayed with sucrose were also more resistant to *M*. *oryzae* infection [6]. Transgenic rice overexpressing PRms, a PR protein from maize seeds, contained higher levels of sucrose in their leaves and were more resistant to fungal and bacterial infection [6]. Transgenic tobacco overexpressing PRms were also more resistant to several fungal pathogens and accumulated higher levels of sucrose in leaf tissues [7]. Along this line of thinking, one would expect plants with increased levels of energy (ATP and sucrose) to exhibit a better growth performance and likely enhanced disease resistance.

To initiate an immune response, plant trans-membrane pattern recognition receptors (PRRs) recognize microbes by their pathogen-associated molecular patterns (PAMPs), such as bacterial flagellin and EF-Tu [8, 9], which results in PAMP-triggered immunity (PTI) protecting these plants against further colonization [10]. The PRRs for bacterial flagellin (FLS2) and EF-Tu (EFR) are RLKs [10], which belong to the largest group, consisting of over 200 genes in the *A*. *thaliana* genome [11]. The second largest group of PRRs are the receptor-like proteins (RLPs), transmembrane receptor-like proteins lacking a cytoplasmic kinase domain (LRR-RLPs) [11]. Of the RLPs, 57 members have been identified in the *A*. *thaliana* genome and have been shown to play a role not only in disease resistance but also in plant development [11]. Virulent pathogens secrete effectors to overcome PTI, leading to effector-triggered susceptibility (ETS). In turn, plants have developed disease resistance proteins (R-proteins) to recognize these pathogen effectors and trigger effector-triggered immunity (ETI) [12]. The majority of the R-genes of *A*. *thaliana* encode cytoplasmic NLR resistance proteins. Based on the presence of an N-terminal Coiled-coil (CC) or Toll/interleukin receptor domain, they can be divided into CC-NLRs and TIR-NLRs, respectively [13].

Over-expression (OE) lines of the *A*. *thaliana* purple acid phosphatase 2 gene (AtPAP2) contain elevated concentrations of ATP and sugars, particularly sucrose [14–16]. The OE lines grow faster and produce 40–57% more seeds at maturity level than do WT plants [15]. AtPAP2 is dually targeted to the outer membranes of both chloroplasts and mitochondria and plays a role in importing protein into these two organelles [17–19]. The over-expression of AtPAP2 thus affects the physiology of these two energy-generating organelles and promotes photosynthesis, sucrose synthesis and ATP production [16]. Microarray studies showed that in the OE lines, the expression of a large number of defense-related genes is suppressed compared to WT [20]. The changes in the expression of nuclear genes in the OE lines were not directly caused by the activity of AtPAP2 but were indirectly caused by the high energy status of the plant cells. In this study, infection assays were carried out to examine whether plants with increased levels of ATP and sucrose were more resistant or susceptible to a bacterial pathogen. In addition, the involvement of SA and JA in the regulation of fitness costs was studied.

## Materials and methods

### Plant materials and growth conditions

In this study, *Arabidopsis thaliana* ecotype Columbia (Col-0) was used as an experimental control. The seeds of transgenic lines OE7, OE21, and T-DNA (*pap2*) along with WT were sown in sterile soil, and 10-day-old seedlings were transplanted in separate pots in the greenhouse or in growth chambers. In the greenhouse, a regime of 50–80% humidity, 21/19°C day/night temperature and 16/8 hours light/dark photoperiod was maintained. The climate settings in the growth chambers were for a 10/14 hours light/dark photoperiod at 21/19°C day/night temperature with a relative humidity of 70%. In the middle of the day, the sucrose and ATP contents of the OE lines were 30–80% and 30–50% higher than those of WT, respectively. In contrast, the sucrose and ATP contents of the *pap2* line were not significantly different from that of the WT [15, 16, 21].

### Microarray analysis

Microarray analyses were performed and reported previously (GEO accession number: GSE40307) [20]. *Arabidopsis* leaves were harvested in the middle of the day and ground in liquid nitrogen. Total RNA was extracted using the RNeasy Mini Kit (Qiagen, USA) and quantified using the Bioanalyzer 2100 (Agilent Technologies, USA). We used 10 μg of total RNA as starting material to synthesize first strand cDNA with an oligo dT primer. Double-stranded DNA synthesis and Cy3 labeling were carried out by NimbleGen Systems, Inc. (USA) in three biological replicates. A standard quantile normalization matrix and the robust multichip average (RMA) algorithm were used for data normalization. The paired Student’s t-test was performed to identify differentially expressed genes in AtPAP2 OE lines compared to WT with a set of log-transformed data. The microarray data of the CC-NLR, TIR-NLR (collectively called NLRs), RLK and RLP protein families were retrieved from the dataset (S1 Table).

### Growth of pathogenic bacteria and fungi for Arabidopsis infection assays

The biotrophic pathogen *Pseudomonas syringae* pv. *tomato (Pst)* strain DC3000 and its mutants *Pst* DC3000 AvrRpm1, *Pst* DC3000 AvrRpt2, *Pst* DC3000 AvrRps4, *Pst* DC3000 AvrPtoB, and *Pst* DC3000 HrcC were employed for the inoculation of plants [22, 23]. Bacteria were cultured on LA plates for 2 days at 28°C with appropriate antibiotics followed by overnight incubation at 28°C in Kings’ B medium with appropriate antibiotics. We added 100 μg/ml rifampicin to allow the selective growth of WT*Pst* DC3000. For *Pst* DC3000 AvrRps4, *Pst* DC3000 AvrRpm1, *Pst* DC3000 AvrRpt2, and *Pst* DC3000 AvrPtoB, 100 μg/ml rifampicin and 100 μg/ml kanamycin were added to the medium, while 100 μg/ml rifampicin and 20 μg/ml kanamycin were used for *Pst* DC3000HrcC cultures. We inoculated 25-day-old greenhouse-grown or 30-day-old growth chamber-grown plants with approximately 10^8^ cfu/ml (OD_600_ = 0.3A) containing silwet (0.05%v/v) until droplet run off.

### Biomass quantification of *Pst DC3000*

For biomass quantification of *Pst* DC3000 strains in infected plants, qRT-PCR analysis was performed with an ABI7300 PCR machine with the SYBR^®^GREEN core kit (Eurogentec Nederland BV, Maastricht, NL). The above-ground parts of three plants (*Pst* DC3000) were harvested for DNA extraction. The qRT-PCR primers to amplify the *Pst* DC3000 *oprF* (outer membrane porin F precursor) gene and AtRbcL genes were used as described previously (S2 Table) [24].

### qRT-PCR analysis of marker genes in the SA and JA pathways

To measure the expression of the *PR1* and *PDF1*.*2* genes, rosette leaves of three individual plants were harvested in three biological replicates. RNA extraction was carried out by an RNeasy Mini Kit (Qiagen) using a 1 μg RNA sample that was subsequently treated with DNase (Invitrogen). First-strand cDNA was synthesized by M-MLV RT(H^-^) (Promega). The qRT-PCR primers used to amplify the *PR1*, *PDF1*.*2* and *Actin2* transcripts used in this study are listed in S2 Table.

### Quantification of SA and JA contents

The rosettes of 3-week-old WT (Col-0), *pap2* mutant and AtPAP2OE lines (OE-7 and OE-21) were collected without any treatment and analyzed by LC/MS for SA and JA contents. D6-SA and D5-JA were added as internal quantitative standards. The data are means ± SD calculated from four biological replicates (200 mg leaves harvested from 3 independent plants were pooled for each biological replicate). *P< 0.05; **P< 0.01 by Student’s *t*-test.

## Results

### Down-regulation of a large number of LRR-RLP and NB-LRR transcripts in AtPAP2 OE lines

Microarray analyses of leaves of 20-day-old *AtPAP2* OE, *pap2* and WT plants were carried out to study transcriptional changes due to the over-expression of AtPAP2 in *Arabidopsis thaliana* (Table 1) [21]. There were 30361 transcripts presented on the microarray. The transcription of the majority (91%) of LRR-containing receptor kinases (LRR-RLK) remained unchanged in OE lines when compared with WT lines (S1a Table and Table 1), while 29% and 7% of receptor-like proteins (LRR-RLPs) were down-regulated and up-regulated in the OE lines, respectively (S1b Table and Table 1). The transcription of 38% of *CC-NLR* genes (S1c Table and Table 1) and 38% of the *TIR-NLR* genes (S1d Table and Table 1) were down-regulated in the OE lines compared to the WT, while the transcription of only 8% of the *CC-NLR* and 6% of the *TIR-NLR* genes were up-regulated, respectively. The OE lines showed decreased transcript accumulation for *NB-LRR*s and *LRR-RLP*s, but not for *LRR-RLK*s. In addition, the expression of *SNC1*, four *SNC1*-like genes and the genes downstream of *SNC1*, including *EDS1*, *EDS5* and *MOS2*, was significantly down-regulated in the OE lines (S1e Table and Table 1). In contrast, the expression levels of most *PR* genes (80%) in uninfected OE lines were similar to those of WT, while 11% were up-regulated and 9% were down-regulated. (S1f Table and Table 1).

**Table 1:** Expression patterns of various types of *R* genes and defense-related genes^1^.

*Genes*	*Total number*	*Unchanged*	*Up-regulated*	*Down-regulated*
***LRR-RLK***	239	219 (91%)	4(2%)	16 (7%)
***LRR-RLP***	55	35(64%)	4(7%)	16(29%)
***CC-NLR***	60	32(53%)	5(8%)	23(38%)
***TIR-NLR***	113	63(56%)	7(6%)	43(38%)
***PR***	44	34(80%)	5(11%)	4(9%)
***SNC1-RPP1***	64	42(66%)	4(6%)	18(28%)

^1^ Samples were collected from WT, OE7 and OE21 lines in three biological replicates. Only the signal intensities in both OE lines are significantly different (*P values*< 0.05 in Student’s t-test) from that of WT; > 1.5-fold was regarded as up-regulated, < 0.67-fold was regarded as down regulated.

### Enhanced susceptibility of OE lines to *Pseudomonas syringae* pv. *tomato*

**Fig 1:**
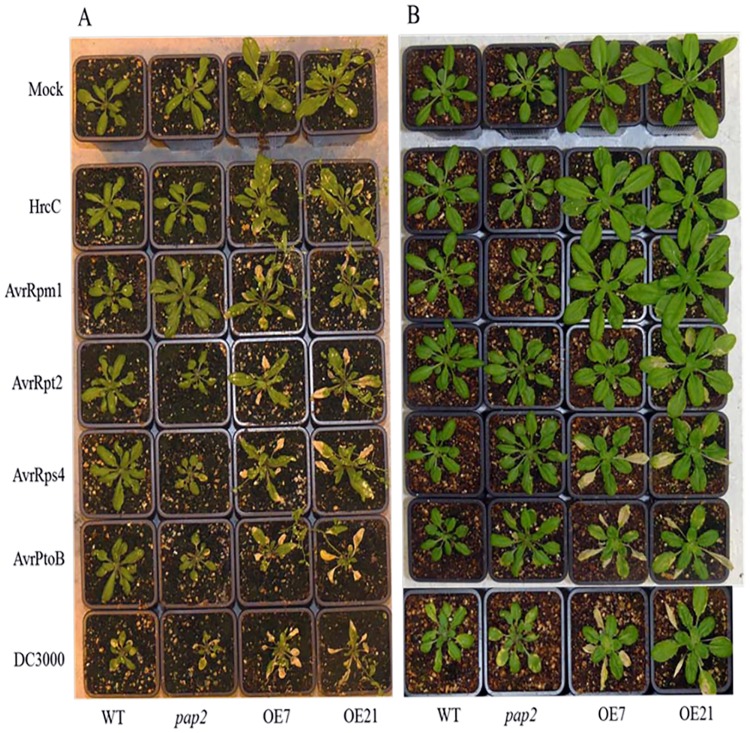
AtPAP2 OE *Arabidopsis thaliana* lines display enhanced susceptibility to *Pseudomonas syringae* pv. *tomato* (Pst) strains. (a) Phenotypes of WT, *pap2*, OE7 and OE21 *A*. *thaliana* lines at 5 dpi with *Pst* DC3000strains carrying different *Avr* genes in greenhouse conditions (16/8 hr light/dark). (b) Phenotype of WT, *pap2*, OE7 and OE21 *A*. *thaliana* lines at 5d pi with *Pst* DC3000 in growth chamber conditions (10/14 hr light/dark). We inoculated 25 day-day-old plants (greenhouse) and 30-day-old plants (growth chamber).

**Fig 2:**
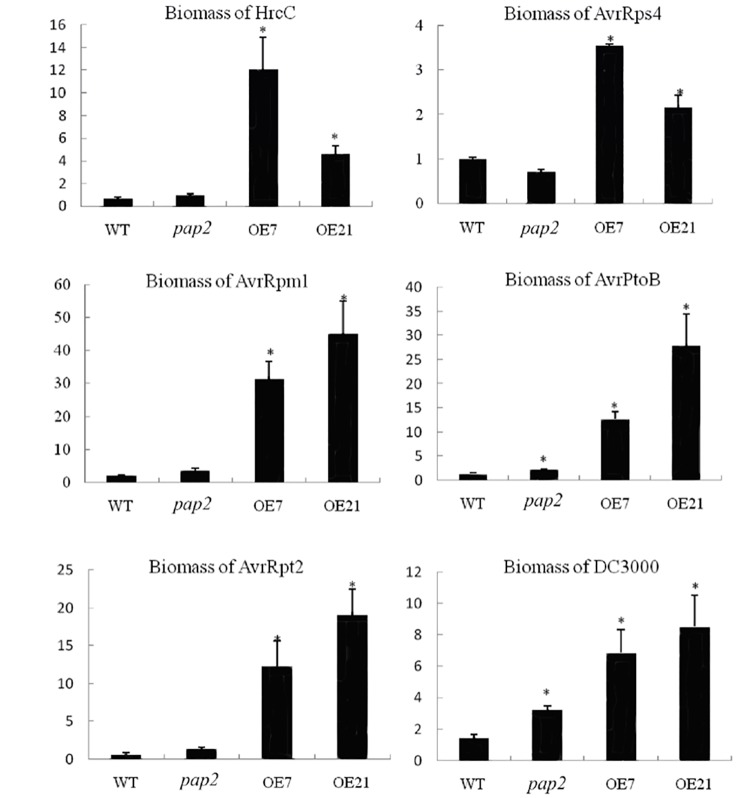
Biomass quantification of the *Pseudomonas syringae* pv. *tomato* DC3000 strains (Pst). DNA was extracted from inoculated plants grown in greenhouse conditions (16/8 hr light/dark) at 5 dpi, and the bacterial biomass was quantified using qPCR on the *oprF* gene. Bacterial biomass was normalized to the amplicon of AtRuBisCO, and its amount in the WT plants was normalized as 1 in each panel. DC3000: (Pst) strain DC3000; AvrRpm1: Pst DC3000 carrying AvrRpm1; AvrRpt2: Pst DC3000 carrying AvrRpt2, AvrRps4: Pst DC3000 carrying AvrRps4; AvrPtoB: Pst DC3000 carrying AvrPtoB (*P< 0.01).

**Fig 3:**
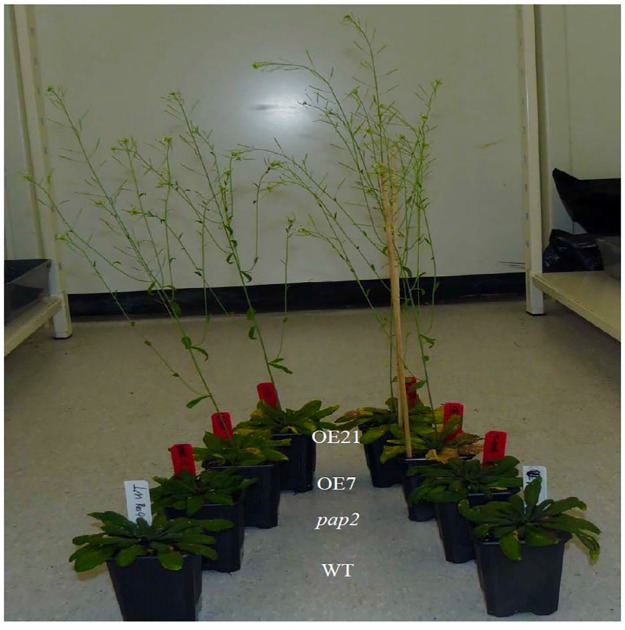
Recovery of infected plants after inoculation with *P*. *syringae* AvrRps4. Photos were taken from 58-day-old plants grown under short day (10/14 hr light/dark photoperiod).

As reflected by the microarray data, the basal mRNA level of the *RPM1* and *RPS2* genes in the OE lines was similar to WT, while the basal mRNAs of *RPS5* (54–62% of WT, *P*<0.005) and *RPS4* (37–58% of WT, *P*<0.005) were downregulated in the OE lines (S1e Table). This situation provided an opportunity to assay whether changes in *R* gene expression would affect the level of resistance against particular pathogens. The pathogenic bacterial strains, *Pseudomonas syringae* pv. *tomato (Pst)* DC3000, *Pst* DC3000 carrying AvrRpm1, *Pst* DC3000 carrying AvrRpt2, and *Pst* DC3000 carrying AvrRps4, were selected to perform inoculation assays (Fig 1). *Pst* DC3000 carrying AvrPtoB was chosen as another virulent strain because its corresponding R gene was not present in *A*. *thaliana* ecotype Columbia (Col-0) [25]. *Pst* DC3000 HrcC mutant was also used as a negative control for its inability to secrete TTSS effectors [26]. To quantify the biomass of Pst DC3000 accumulation in the four lines after infection, q-RT-PCR analysis was performed at 5 days postinoculation (dpi) under controlled greenhouse conditions (Fig 2). We detected increased *Pst* DC 3000 biomass in inoculated OE lines compared to WT and *pap2* lines, suggesting that this bacterial pathogen proliferates better in plants with increased levels of ATP and sucrose. Interestingly, although the OE lines were more susceptible to *P*. *syringae pv*. *tomato*, they recovered and grew more rapidly than the WT after inoculation (Fig 3).

The results obtained from the inoculation assays showed that OE lines were more susceptible than were WT and *pap2* lines upon infection with the *Pst* strain DC3000, *Pst* DC3000 carrying AvrRpm1, *Pst* DC3000 carrying AvrRpt2, or *Pst* DC3000 carrying AvrRps4 (Figs 1A and 2). Effector-triggered signaling takes place upon recognition by specific *R* genes, such as *AvrRpm1-RPM1* [27, 28], *AvrRpt2-RPS2* [29] or *AvrRps4-RPS4* [30]. Comparing the WT and OE lines, the basal transcription levels of RPS5 (54–62% of WT, p<0.005) and RPS4 (37–58% of WT, P<0.005) were lower (S1e Table), consistent with the observed increase in the susceptibility phenotypes of these lines when compared to the WT and the *pap2* mutant. However, the basal transcription levels of RPM1 and RPS2 in the OE lines showed no significant difference from the WT. Therefore, these data suggest that the basal levels of these R genes in the OE lines were not the sole determinants of their enhanced disease susceptibility. The OE lines were also more susceptible to *Pst* DC3000 carrying AvrPtoB (Figs 1A and 2) as the *Pto* gene is not present in the four lines, and thus this strain was expected to behave like *Pst* DC3000, which lacks any *Avr* genes. Similar results were also obtained for plants grown in a growth chamber (10/14 hr light/dark photoperiod) at 5 dpi, although increased susceptibility under this growth condition was generally not as pronounced as that under greenhouse conditions (16/8 hr light/dark photoperiod) (Fig 1B). Overall, the OE lines were more susceptible to all tested *P*. *syringae* strains compared to WT and *pap2* lines. Hence, the increased susceptibility of the OE lines to *Pst* DC3000 strains cannot be solely attributed to the suppressed expression of R-genes and specific *R* gene-mediated defense signaling (AvrRpm1-RPM1, AvrRpt2-RPS2 or AvrRp4-RPS4) but could be due to the down-regulation of genes downstream of R-proteins, such as *SNC1*, *MOS2*, *EDS1*, *EDS5*, *NDR1*, and others (S1e Table).

### Effects of high energy supply on the SA- and JA-dependent defense pathways

*Pseudomonas syringae* pv. *tomato* is a biotrophic pathogen, and resistance of *A*. *thaliana* to this pathogen is salicylic (SA)-dependent. Jasmonic acid (JA) is also an important defense hormone that can antagonize the effects of SA [31, 32]. The SA-dependent defense pathway is initiated upon recognition of pathogens maintaining a biotrophic lifestyle, whereas the JA-dependent pathways are deployed against necrotrophs [33]. The activation of *PR* gene expression is associated with the SA pathway, while the deployment of the JA-dependent pathway is associated with the expression of plant defensins (PDFs) [34]. Genetic studies have provided a series of mutants with overproduction of either SA (e.g., *cpr1*, *cpr5*, *cpr6*, *dnd1*, *dnd2*, etc.), JA or ethylene (e.g., *cev1*) [35–39]. It was observed that the elevated resistance of these mutants to pathogens was generally associated with increased levels of PR and PDF defense proteins, leading to reduced fitness (e.g., stunted growth, reduced rosette leaves, etc.).

**Fig 4:**
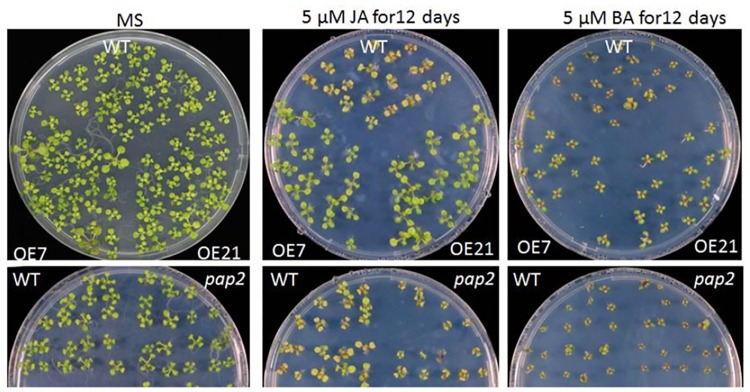
Sensitivity of *Arabidopsis thaliana* seeds of WT, *pap2* and overexpression lines OE7 and OE21 to jasmonic acid (JA) and benzoic acid (BA). Seeds of OE lines along with *pap2* and WT were germinated on MS or MS supplemented with 5 μM JA or 5 μM BA for 12 days. Note that OE lines are more resistant to JA treatment than WT, whereas the *pap2* line was more susceptible than the WT.

**Fig 5:**
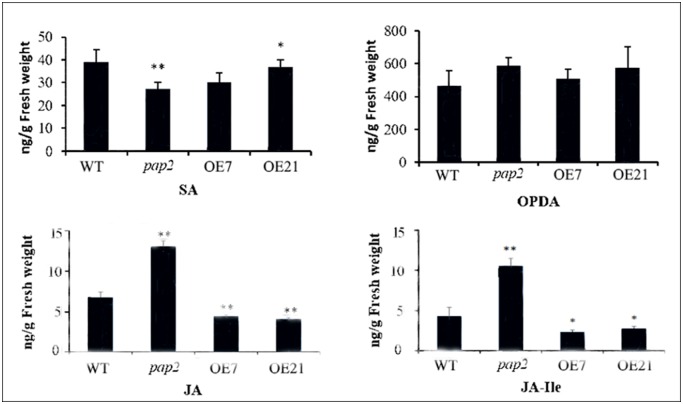
SA and JA levels in leaves of 3-week-old *Arabidopsis thaliana* plants. Leaves were collected from 3-week-old WT (Col-0), *pap2* mutant and OE lines (OE-7 and OE-21) plants. SA, JA, JA conjugated with isoleucine (JA-Ile) and JA precursor 12-oxo-phytodienoic acid (OPDA) levels were determined by LC/MS. D6-SA and D5-JA were added as internal standards. *P< 0.05; **P< 0.01 by Student’s *t*-test.

To study whether the SA and JA defense pathways in the AtPAP2 OE lines were altered, the seedlings of transgenic lines along with *pap2* and WT were treated with benzoic acid (BA) and JA. As shown in Fig 4, the OE lines were more resistant to JA treatment than was the WT, whereas the *pap2* line was more sensitive than the WT. The levels of SA, JA, JA conjugated with isoleucine (JA-Ile) and JA precursor 12-oxo-phytodienoic acid (OPDA) in 3-week-old plants were analyzed by LC/MS (Fig 5). While there was no difference in the levels of OPDA between the lines, the levels of JA and its derivative JA-Ile were significantly higher in the *pap2* line than the WT, whereas their levels were significantly lower in the OE lines than in the WT, indicating that the conversion of OPDA to JA was suppressed in the OE lines or the breakdown of JA was more rapid in the OE lines. While the OE lines contained less endogenous JA, they were more resistant to exogenous JA treatment (Fig 4). In contrast, the endogenous SA levels in the OE lines was not different from those of the WT, and their sensitivity to exogenous SA treatment was also similar to WT.

**Fig 6:**
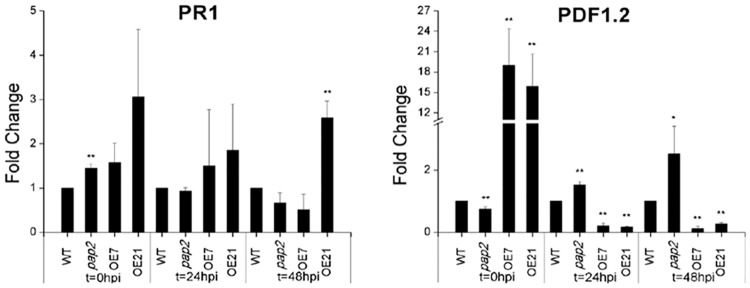
Relative expression of SA and JA defense pathway marker genes *PR1* and *PDF1*.*2*, respectively, in the four lines (WT, *pap2*, OE7 and OE21) were determined by qRT-PCR at three time points (t = 0 hpi, t = 24 hpi and t = 48 hpi) after *Pseudomonas syringae* pv. *tomato* DC3000 inoculation. Samples were collected in three biological replicates (**P< 0.01), and the expression level in WT at each time point was set to 1 1 for normalization.

To further characterize the expression of the two marker genes within the SA and the JA signaling pathways, the expression of *PR1* and *PDF1*.*2* were quantified using qRT-PCR [34, 40]. Samples were collected at 0 hours post inoculation (hpi), 24 hpi and 48 hpi with *Pst* DC3000. *PDF1*.*2* expression was increased in the OE lines compared to the WT and *pap2* lines before infection (Fig 6). At both 24 hpi and 48 hpi, the expression of *PDF1*.*2* in OE lines was significantly lower than in WT. However, its expression in the *pap2* line was up-regulated at 24 hpi and 48 hpi. These data show that AtPAP2 over-expression lowers basal JA contents in uninfected leaves of AtPAP2 OE lines, which in turn results in a lower expression of *PDF1*.*2* after inoculation with a biotrophic pathogen. In contrast, *PR1* expression in the OE lines at 0 hpi was similar to WT, which is consistent with the results of the microarray data (Fig 6 and S1f Table). At 24 hpi, the *PR1* expression in the OE lines was similar to that in the WT and *pap2* lines (Fig 6).

## Discussion

### Arabidopsis with high endogenous sucrose is not necessarily more resistant to pathogen

The perception that sucrose acts as a signaling molecule in plant defense was supported by experiments treating plants with exogenous sucrose [4–7]. Hypotonic feeding of rice seedlings with 300 mM sucrose was shown to enhance PR gene expression, and the seedlings sprayed with 300 mM sucrose were also more resistant to *M*. *oryzae* infection [6]. However, our data showed that transgenic lines with higher endogenous sucrose are more susceptible to *P*. *syringae* infection, which is opposite to the above perception. The contradicting outcomes could arise from the opposite signals or impacts induced by exogenous and endogenous sucrose. Exogenous sucrose is well-known to induce the transcription of enzymes in the anthocyanin production pathway [41]; however, the abundance of most of these transcripts was significantly lower in the AtPAP2 OE lines before sucrose treatment, and the OE lines produced significantly lower anthocyanin after sucrose treatment [20]. As exogenous sucrose treatment is not a natural condition in nature, the conclusions derived from experimental data generated from exogenous sucrose treatment should be interpreted with caution. Therefore, the AtPAP2 OE lines will be a valuable tool to study the impact of high endogenous sucrose/energy status on the immune defense of plants against different types of pathogens and modes of infections.

### Impact of high energy status on the transcription of *PRR* and *R* genes

By studying the microarray data of two independent AtPAP2 OE lines and those of WT, the expression of the *RLK*, *RLP*, *NLR* and *PR* genes could be compared. Our data showed that the high ATP and sucrose levels of the OE lines did not affect the transcription of *RLKs* that could maintain their role in PAMP detection. In contrast, the transcription of *RLP* and *NLR* genes was significantly suppressed in the OE lines (Table 1). While the transcription of only 8% of CC-NLR and 6% of TIR-NLR genes was up-regulated, both NB-LRR classes of R-genes showed 38% down-regulation in the OE lines when compared to the WT (Table 1). In contrast, the transcription of 80% of the *PR* genes was unaltered. This result is not surprising as most *PR* genes (e.g., *PR1* and *PDF2*.*1*) are only induced by infection in WT plants. After infection, PR proteins can constitute up to 1% of the total soluble leaf proteins [42], which is an energy-requiring process that should only be triggered when needed. Consequently, energy expenditure for cell defense and plant resistance can be kept at a low level.

### Effects of high energy supply on the SA- and JA-dependent defense pathways

SA actively contributes to resistance against *P*. *syringae* pv. *tomato* in *A*. *thaliana* during *NLR* gene-mediated defense signaling [43], and antagonism between the SA and JA pathway has been suggested [44]. However, lower concentrations of SA (8–350 μM) and JA (10–100 μM) were shown to synergistically activate the expression of *PR1* and *PDF1*.*2* in wild type *A*. *thaliana* and tobacco [45]. We therefore examined whether the SA and JA defense pathways are altered in the AtPAP2 OE lines. The basal levels of SA and OPDA were unaltered in the OE lines, but the levels of JA and JA-Ile were substantially lower in the OE lines than in the WT (Fig 5). In the JA synthesis pathway, OPDA is synthesized from linolenic acid in chloroplasts and then transported to the peroxisome, for conversion into JA by three consecutive enzymatic steps [46]. The lower JA level in the OE lines could be due to a reduction of JA synthesis in peroxisomes after the production of OPDA in chloroplasts or due to a higher JA degradation rate in uninfected leaf cells. Paradoxically, the seedlings of the OE lines with lower endogenous JA were more resistant to exogenous JA treatment than was the WT (Fig 4). In contrast, the endogenous levels of JA and JA-Ile were significantly higher in the *pap2* line than in WT, but the *pap2* line was more sensitive to exogenous JA treatment. Hence, our results showed that in the OE and *pap2* lines, the JA defense pathway is significantly affected.

JA treatment is known to induce transcription of *PDF1*.*2* in *A*. *thaliana*. As the OE lines contained lower JA levels prior to infection compared to WT, a lower expression of *PDF1*.*2* in the OE lines was expected. However, qRT-PCR showed that the transcription of *PDF1*.*2* was significantly enhanced in the OE lines (OE7 and OE21), while the *PDF1*.*2* expression in the *pap2* line was down-regulated (Fig 6). In contrast, no significant difference in the basal expression of the *PR1* gene, a downstream defense gene in the SA pathway, was observed between the WT and OE lines. Upon inoculation with *Pst* DC3000 (t = 24 hpi and t = 48 hpi), the expression of *PDF1*.*2* was significantly reduced in the OE lines when compared to the WT and *pap2* line, which was not observed for *PR1* expression. Hormonal actions on plant immune responses are therefore regulated by complex signaling networks [45, 47].

The significantly increased JA content in *pap2* but decreased JA content in OE lines prior to *Pst* DC3000 infection suggests that the over-expression of AtPAP2 suppresses JA biosynthesis, but the disruption of AtPAP2 promotes JA biosynthesis (Fig 5). A significantly lower SA content in *pap2* was also noted. Hence, an antagonistic basal level of SA and JA was observed in *pap2*. However, the changes in the basal levels of SA and JA in *pap2* were not sufficient to significantly affect its susceptibility toward *Pst* infection. Instead, the basal sucrose and ATP contents of *pap2* were similar to those of WT, and both lines exhibited similar susceptibility to *Pst* strains. Hence, our observation that Arabidopsis with higher levels of ATP and sucrose are more susceptible to *P*. *syringae* is still valid.

### Mechanisms underlying the increased susceptibility of the high energy plants to *P*. *syringae* pv. *tomato* infection

The transcriptional levels of all types of *NLR* genes were reported in the microarray data (S1 Table). They include *RPM1* (no change in OE lines), which confers resistance to *Pst* DC3000 carrying avirulence (*Avr*) effector *AvrRpm1*, *RPS2* (no change), which confers resistance to *Pst* DC3000 carrying *AvrRpt2*, and *RPS4* (down-regulated), which confers resistance to *Pst* DC3000 carrying *AvrRPS4* (S1 Table). Infection assays were performed using these bacterial strains together with *Pst* DC3000, *Pst* DC3000 (*AvrPtoB*), which does not have a corresponding *NLR* gene in *A*. *thaliana*, and *Pst* DC3000 (*HrcC*), which is defective for TTSS (Fig 2). The general enhanced susceptibility of OE lines toward these strains compared to WT implies that transcriptional changes in the *NLR* and *RLP* genes are not the sole determining factor for the increased susceptibility of the OE lines (S1 Table) [48, 49]. After the activation of R genes in cytosol, downstream pathways are required to transmit the signals to the nucleus to initiate the immune response [49]. For the activation of immune responses, the CC-NLRs require Non-race-specific disease resistance (*NDR1*) and other accessory proteins, whereas TIR-NLRs require *enhanced disease susceptibility 1* (*EDS1*), *phytoalexin deficient 4* (*PAD4*) and s*enescence associated gene 101* (*SAG101*) [50, 51]. EDS1 and PAD4 are defense components that shuttle between the nucleus and the cytoplasm [49]. EDS1 forms complexes with PAD4 in both compartments, and EDS1 forms complexes with SAG101 in the nuclear compartment [51]. The SA pathway was shown to play key roles in the major cell defense pathway against *P*. *syringae* pv. tomato in plants. While our data showed that the high energy status in the OE lines did not affect the basal level of SA in plants, some genes required for SA induction, including *EDS1* and *EDS5*, were downregulated in the OE lines [32]. *EDS1* is required for SA production, PR1 expression and resistance to *P*. *syringae* [47]. It also acts as a negative regulator of ET/JA defense signaling and a repressor of PDF1.2 induction by MeJA [32]. Microarray data showed that the transcription levels of *NDR1*, *EDS1* and *EDS5* were suppressed by 19–28%, 61–63% and 38–46% in the two OE lines, respectively (S1e Table). Their suppression may have a negative effect on the resistance to *P*. *syringae* strains in the OE lines.

Another TIR-NLR type of *R*-gene that is significantly suppressed in the OE lines (12–15% of WT) is *SNC1 (suppressor of NPR1*, *constitutive 1)*. SNC1 binds to TPR1 transcriptional co-repressor, which in turn represses the expression of several negative regulators of defense responses [48], including two negative regulators of plant innate immunity, defense no death 1 (DND1) and defense no death 2 (DND2) [39]. SA induces the expression of *SNC1* [52], and over-expression of *SNC1* enhances disease resistance to *Pst* DC3000 but suppresses plant growth [53]. The lower basal level of *SNC1* in the OE lines may therefore potentiate them to be more susceptible to infection with *P*. *syringae* pv. *tomato* strains. A *snc1* suppressor screen identified a number of *modifiers of snc1* (mos) mutants that may play roles in nucleo-cytoplasmic trafficking, transcriptional regulation/RNA processing, and protein modification [49], of which the transcription of a RNA-binding protein, *MOS2*, was strongly suppressed in the two OE lines (40% of WT) [54]. In addition to *SNC1*, there are a few *SNC1-like* genes in the genome of *A*. *thaliana*, and the transcription of four *SNC1-like* genes in both OE lines was only 3%, 22 31%, 27–31% and 41–47% of that of the WT (S1e Table). They, together with *SNC1*, belong to the TNL-E subclass of the *TIB-NLR* gene family [55]. The functions of these four *SNC1-like* genes are still unknown, and whether they play differential roles or share the same downstream signaling pathway is a subject of further studies. The down-regulation of the transcription of these *SNC1-like* genes, *RLP*, *NLR* and *R*-genes in the downstream defense signaling pathways may explain the increased susceptibility of the OE lines to various *P*. *syringae* strains. In the Arabidopsis genome, there are 60 and 115 CC-NLR and TIB-NLR genes, respectively. While they may be responsive toward various Avr proteins, it is likely that convergence of different downstream signaling pathways acts together to turn on these defense genes. The downregulation of the mRNA expression of *SNC1*/*MOS2*/*EDS1/EDS5* and 4 *SNC1*-like genes in both OE lines in the uninfected state could be a reason why the OE lines are less resistant to various *P*. *syringae* strains. The lower expression of these genes may cause a slower immune response in OE lines and allow the bacteria to have more time to replicate and accumulate before an immune response is mounted.

### The trade-off between growth and disease resistance

To effectively defend themselves against pathogen infection, plants usually sacrifice some growth. A collection of mutants with the overproduction of either SA or JA exhibited compromised plant fitness [35–39], supporting the hypothesis that active defense correlates with delayed growth. The AtPAP2 OE lines contain elevated levels of ATP, malate, citrate, fumarate and sucrose [20] and grow faster than WT [16]. The extra energy in the OE lines could have been deployed for the growth of the plants, and the momentum of carbon metabolism shifts to sucrose synthesis and ATP production. Disease resistance was weakened as reflected by the suppression of the basal mRNA expression levels of some RLPs, NLRs, and downstream genes (e.g., *SNC1*, *EDS1*, *EDS5*, *MOS2*) (Table 1). This effect reflects the fitness cost tradeoff between growth and disease resistance. In this study, *Arabidopsis thaliana* with high fitness was found to be more susceptible to a biotrophic bacterial pathogen. Further studies on its susceptibility toward other pathogens, such as biotrophic fungi and necrotrophic pathogens, will provide more information about the relationship between plant fitness and disease resistance.

## Supporting information

S1 Table Microarray data. (XLSX)

S2 Table Primers used in this study. (DOCX)

## References

[1] Vila-Aiub MM, Gundel PE, Preston C. Experimental Methods for Estimation of Plant Fitness Costs Associated with Herbicide-Resistance Genes. Weed Sci. 2015;63:203–16.

[2] Primack RB, Kang H. Measuring Fitness and Natural-Selection in Wild Plant-Populations. Annu Rev Ecol Syst. 1989;20:367–96.

[3] Tian D, Traw MB, Chen JQ, Kreitman M, Bergelson J. Fitness costs of R-gene-mediated resistance in Arabidopsis thaliana. Nature. 2003;423(6935):74–7. 10.1038/nature01588 12721627

[4] Tauzin AS, Giardina T. Sucrose and invertases, a part of the plant defense response to the biotic stresses. Front Plant Sci. 2014;5:293 10.3389/fpls.2014.00293 25002866 PMC4066202

[5] Thibaud MC, Gineste S, Nussaume L, Robaglia C. Sucrose increases pathogenesis-related PR-2 gene expression in Arabidopsis thaliana through an SA-dependent but NPR1-independent signaling pathway. Plant Physiol Biochem. 2004;42(1):81–8. 10.1016/j.plaphy.2003.10.012 15061088

[6] Gomez-Ariza J, Campo S, Rufat M, Estopa M, Messeguer J, San Segundo B, et al Sucrose-mediated priming of plant defense responses and broad-spectrum disease resistance by overexpression of the maize pathogenesis-related PRms protein in rice plants. Mol Plant Microbe Interact. 2007;20(7):832–42. 10.1094/MPMI-20-7-0832 17601170

[7] Murillo I, Roca R, Bortolotti C, Segundo BS. Engineering photoassimilate partitioning in tobacco plants improves growth and productivity and provides pathogen resistance. Plant J. 2003;36(3):330–41. 14617090 10.1046/j.1365-313x.2003.01880.x

[8] Kunze G, Zipfel C, Robatzek S, Niehaus K, Boller T, Felix G. The N terminus of bacterial elongation factor Tu elicits innate immunity in Arabidopsis plants. Plant Cell. 2004;16(12):3496–507. 10.1105/tpc.104.026765 15548740 PMC535888

[9] Gomez-Gomez L, Boller T. FLS2: an LRR receptor-like kinase involved in the perception of the bacterial elicitor flagellin in Arabidopsis. Mol Cell. 2000;5(6):1003–11. Epub 2000/07/27. 10911994 10.1016/s1097-2765(00)80265-8

[10] Nicaise V, Roux M, Zipfel C. Recent advances in PAMP-triggered immunity against bacteria: pattern recognition receptors watch over and raise the alarm. Plant Physiol. 2009;150(4):1638–47. Epub 2009/06/30. 10.1104/pp.109.139709 19561123 PMC2719144

[11] Wang GD, Ellendorff U, Kemp B, Mansfield JW, Forsyth A, Mitchell K, et al A genome-wide functional investigation into the roles of receptor-like proteins in Arabidopsis. Plant Physiol. 2008;147(2):503–17. 10.1104/pp.108.119487 18434605 PMC2409048

[12] Jones JDG, Dangl JL. The plant immune system. Nature. 2006;444(7117):323–9. 10.1038/nature05286 17108957

[13] Meyers BC, Kozik A, Griego A, Kuang H, Michelmore RW. Genome-wide analysis of NBS-LRR-encoding genes in Arabidopsis. Plant Cell. 2003;15(4):809–34. Epub 2003/04/03. 10.1105/tpc.009308 12671079 PMC152331

[14] Liang C, Liu X, Sun Y, Yiu SM, Lim BL. Global small RNA analysis in fast-growing Arabidopsis thaliana with elevated concentrations of ATP and sugars. Bmc Genomics. 2014;15:116 10.1186/1471-2164-15-116 24507710 PMC3925372

[15] Sun F, Suen PK, Zhang Y, Liang C, Carrie C, Whelan J, et al A dual-targeted purple acid phosphatase in Arabidopsis thaliana moderates carbon metabolism and its overexpression leads to faster plant growth and higher seed yield. The New phytologist. 2012;194(1):206–19. Epub 2012/01/25. 10.1111/j.1469-8137.2011.04026.x 22269069

[16] Liang C, Zhang Y, Cheng S, Osorio S, Sun Y, Fernie AR, et al Impacts of high ATP supply from chloroplasts and mitochondria on the leaf metabolism of *Arabidopsis thaliana*. Front Plant Sci. 2015;6. 26579168 10.3389/fpls.2015.00922 PMC4623399

[17] Law YS, Zhang R, Guan X, Cheng S, Sun F, Duncan O, et al Phosphorylation and Dephosphorylation of the Presequence of pMORF3 During Import into Mitochondria from Arabidopsis thaliana. Plant Physiol. 2015;169:1–12. 26304849 10.1104/pp.15.01115 PMC4587475

[18] Zhang R, Guan X, Law YS, Sun F, Chen S, Wong KB, et al AtPAP2 modulates the import of the small subunit of Rubisco into chloroplasts. Plant signaling & behavior. 2016;11(10):e1239687. 27700374 10.1080/15592324.2016.1239687 PMC5117095

[19] Sun F, Carrie C, Law S, Murcha MW, Zhang R, Law YS, et al AtPAP2 is a tail-anchored protein in the outer membrane of chloroplasts and mitochondria. Plant signaling & behavior. 2012;7(8):927–32. Epub 2012/07/04. 22751362 10.4161/psb.20769 PMC3474687

[20] Sun F, Liang C, Whelan J, Yang J, Zhang P, Lim BL. Global transcriptome analysis of AtPAP2—overexpressing Arabidopsis thaliana with elevated ATP. Bmc Genomics. 2013;14:752 Epub 2013/11/05. 10.1186/1471-2164-14-752 24180234 PMC3829102

[21] Sun F, Liang C, Whelan J, Yang J, Zhang P, Lim BL. Global transcriptome analysis of AtPAP2-overexpressing Arabidopsisthaliana with elevated ATP. Bmc Genomics. 2013;14. 10.1186/1471-2164-14-752 PMC3829102 24180234

[22] Crute I, Beynon J, Dangl J, Holub E, Mauch-Mani B, Slusarenko A, et al Microbial pathogenesis of Arabidopsis. 1994;27:705–47.

[23] Katagiri F, Thilmony R, He SY. The Arabidopsis thaliana-pseudomonas syringae interaction. Arabidopsis Book. 2002;1:e0039 Epub 2002/01/01. 10.1199/tab.0039 22303207 PMC3243347

[24] Brouwer M, Lievens B, Van Hemelrijck W, Van den Ackerveken G, Cammue BPA, Thomma BPHJ. Quantification of disease progression of several microbial pathogens on Arabidopsis thaliana using real-time fluorescence PCR. Fems Microbiol Lett. 2003;228(2):241–8. 14638430 10.1016/S0378-1097(03)00759-6

[25] Lin NC, Martin GB. Pto- and Prf-mediated recognition of AvrPto and AvrPtoB restricts the ability of diverse pseudomonas syringae pathovars to infect tomato. Mol Plant Microbe Interact. 2007;20(7):806–15. Epub 2007/07/03. 10.1094/MPMI-20-7-0806 17601168

[26] Roine E, Wei W, Yuan J, Nurmiaho-Lassila EL, Kalkkinen N, Romantschuk M, et al Hrp pilus: an hrp-dependent bacterial surface appendage produced by Pseudomonas syringae pv. tomato DC3000. Proc Natl Acad Sci U S A. 1997;94(7):3459–64. Epub 1997/04/01. 9096416 10.1073/pnas.94.7.3459 PMC20392

[27] Dangl JL, Ritter C, Gibbon MJ, Mur LA, Wood JR, Goss S, et al Functional homologs of the Arabidopsis RPM1 disease resistance gene in bean and pea. Plant Cell. 1992;4(11):1359–69. Epub 1992/11/01. 1477552 10.1105/tpc.4.11.1359 PMC160224

[28] Bisgrove SR, Simonich MT, Smith NM, Sattler A, Innes RW. A disease resistance gene in Arabidopsis with specificity for two different pathogen avirulence genes. Plant Cell. 1994;6(7):927–33. Epub 1994/07/01. 10.1105/tpc.6.7.927 8069104 PMC160489

[29] Mindrinos M, Katagiri F, Yu G.L, and Ausubel F.M. The Arabidopsis thaliana disease resistance gene RPS2 encodes a protein containing a nucleotide-binding site and leucine-rich repeats. Cell. 1994;78:1089–99. 7923358 10.1016/0092-8674(94)90282-8

[30] Hinsch M, Staskawicz B. Identification of a new Arabidopsis disease resistance locus, RPs4, and cloning of the corresponding avirulence gene, avrRps4, from Pseudomonas syringae pv. pisi. Mol Plant Microbe Interact. 1996;9(1):55–61. Epub 1996/01/01. 8589423 10.1094/mpmi-9-0055

[31] Bari R, Jones J. Role of plant hormones in plant defence responses. Plant Mol Biol. 2009;69(4):473–88. 10.1007/s11103-008-9435-0 19083153

[32] Kunkel BN, Brooks DM. Cross talk between signaling pathways in pathogen defense. Curr Opin Plant Biol. 2002;5(4):325–31. 12179966 10.1016/s1369-5266(02)00275-3

[33] Thomma BPHJ, Penninckx IAMA, Broekaert WF, Cammue BPA. The complexity of disease signaling in Arabidopsis. Curr Opin Immunol. 2001;13(1):63–8. 11154919 10.1016/s0952-7915(00)00183-7

[34] Thomma BPHJ, Eggermont K, Penninckx IAMA, Mauch-Mani B, Vogelsang R, Cammue BPA, et al Separate jasmonate-dependent and salicylate-dependent defense-response pathways in Arabidopsis are essential for resistance to distinct microbial pathogens. P Natl Acad Sci USA. 1998;95(25):15107–11. 10.1073/pnas.95.25.15107 PMC24583 9844023

[35] Bowling SA, Clarke JD, Liu YD, Klessig DF, Dong XN. The cpr5 mutant of Arabidopsis expresses both NPR1-dependent and NPR1-independent resistance. Plant Cell. 1997;9(9):1573–84. 10.1105/tpc.9.9.1573 9338960 PMC157034

[36] Clarke JD, Liu YD, Klessig DF, Dong XN. Uncoupling PR gene expression from NPR1 and bacterial resistance: Characterization of the dominant Arabidopsis cpr6-1 mutant. Plant Cell. 1998;10(4):557–69. 9548982 10.1105/tpc.10.4.557 PMC144011

[37] Clough SJ, Fengler KA, Yu IC, Lippok B, Smith RK, Bent AF. The Arabidopsis dnd1 "defense, no death" gene encodes a mutated cyclic nucleotide-gated ion channel. P Natl Acad Sci USA. 2000;97(16):9323–8. 10.1073/pnas.150005697 PMC16866 10900264

[38] Ellis C, Turner JG. The Arabidopsis mutant cev1 has constitutively active jasmonate and ethylene signal pathways and enhanced resistance to pathogens. Plant Cell. 2001;13(5):1025–33. 11340179 10.1105/tpc.13.5.1025 PMC135553

[39] Jurkowski GI, Smith RK, Yu IC, Ham JH, Sharma SB, Klessig DF, et al Arabidopsis DND2, a second cyclic nucleotide-gated ion channel gene for which mutation causes the "defense, no death" phenotype. Mol Plant Microbe In. 2004;17(5):511–20. 10.1094/MPMI.2004.17.5.511 15141955

[40] Spoel SH, Koornneef A, Claessens SMC, Korzelius JP, Van Pelt JA, Mueller MJ, et al NPR1 modulates cross-talk between salicylate- and jasmonate-dependent defense pathways through a novel function in the cytosol. Plant Cell. 2003;15(3):760–70. 10.1105/tpc.009159 12615947 PMC150028

[41] Solfanelli C, Poggi A, Loreti E, Alpi A, Perata P. Sucrose-specific induction of the anthocyanin biosynthetic pathway in Arabidopsis. Plant Physiol. 2006;140(2):637–46. 10.1104/pp.105.072579 16384906 PMC1361330

[42] Van Loon LC. Induced resistance in plants and the role of pathogenesis-related proteins. Eur J Plant Pathol. 1997;103(9):753–65.

[43] Vlot AC, Dempsey DA, Klessig DF. Salicylic Acid, a multifaceted hormone to combat disease. Annu Rev Phytopathol. 2009;47:177–206. Epub 2009/04/30. 10.1146/annurev.phyto.050908.135202 19400653

[44] Flors V, Ton J, van Doorn R, Jakab G, Garcia-Agustin P, Mauch-Mani B. Interplay between JA, SA and ABA signalling during basal and induced resistance against Pseudomonas syringae and Alternaria brassicicola. Plant Journal. 2008;54(1):81–92. 10.1111/j.1365-313X.2007.03397.x 18088307

[45] Mur LA, Kenton P, Atzorn R, Miersch O, Wasternack C. The outcomes of concentration-specific interactions between salicylate and jasmonate signaling include synergy, antagonism, and oxidative stress leading to cell death. Plant Physiol. 2006;140(1):249–62. 10.1104/pp.105.072348 16377744 PMC1326048

[46] Wasternack C, Hause B. Jasmonates and octadecanoids: signals in plant stress responses and development. Prog Nucleic Acid Res Mol Biol. 2002;72:165–221. 12206452 10.1016/s0079-6603(02)72070-9

[47] Brodersen P, Petersen M, Bjorn Nielsen H, Zhu S, Newman MA, Shokat KM, et al Arabidopsis MAP kinase 4 regulates salicylic acid- and jasmonic acid/ethylene-dependent responses via EDS1 and PAD4. Plant J. 2006;47(4):532–46. 10.1111/j.1365-313X.2006.02806.x 16813576

[48] Zhu Z, Xu F, Zhang Y, Cheng YT, Wiermer M, Li X, et al Arabidopsis resistance protein SNC1 activates immune responses through association with a transcriptional corepressor. Proc Natl Acad Sci U S A. 2010;107(31):13960–5. 10.1073/pnas.1002828107 20647385 PMC2922275

[49] Monaghan J, Germain H, Weihmann T, Li X. Dissecting plant defence signal transduction: modifiers of snc1 in Arabidopsis. Can J Plant Pathol. 2010;32(1):35–42.

[50] Aarts N, Metz M, Holub E, Staskawicz BJ, Daniels MJ, Parker JE. Different requirements for EDS1 and NDR1 by disease resistance genes define at least two R gene-mediated signaling pathways in Arabidopsis. P Natl Acad Sci USA. 1998;95(17):10306–11. 10.1073/pnas.95.17.10306 PMC21504 9707643

[51] Feys BJ, Wiermer M, Bhat RA, Moisan LJ, Medina-Escobar N, Neu C, et al Arabidopsis SENESCENCE-ASSOCIATED GENE101 stabilizes and signals within an ENHANCED DISEASE SUSCEPTIBILITY1 complex in plant innate immunity. Plant Cell. 2005;17(9):2601–13. 10.1105/tpc.105.033910 16040633 PMC1197438

[52] Shirano Y, Kachroo P, Shah J, Klessig DF. A gain-of-function mutation in an Arabidopsis Toll Interleukin1 receptor-nucleotide binding site-leucine-rich repeat type R gene triggers defense responses and results in enhanced disease resistance. Plant Cell. 2002;14(12):3149–62. 10.1105/tpc.005348 12468733 PMC151208

[53] Benschop JJ, Mohammed S, O'Flaherty M, Heck AJ, Slijper M, Menke FL. Quantitative phosphoproteomics of early elicitor signaling in Arabidopsis. Molecular & cellular proteomics: MCP. 2007;6(7):1198–214. Epub 2007/02/24. 17317660 10.1074/mcp.M600429-MCP200

[54] Zhang Y, Cheng YT, Bi D, Palma K, Li X. MOS2, a protein containing G-patch and KOW motifs, is essential for innate immunity in Arabidopsis thaliana. Curr Biol. 2005;15(21):1936–42. 10.1016/j.cub.2005.09.038 16271871

[55] Tan X, Meyers BC, Kozik A, West MA, Morgante M, St Clair DA, et al Global expression analysis of nucleotide binding site-leucine rich repeat-encoding and related genes in Arabidopsis. BMC Plant Biol. 2007;7:56 10.1186/1471-2229-7-56 17956627 PMC2175511

