# Supplementary material for: Transgenic Arabidopsis thaliana containing increased levels of ATP and sucrose is more susceptible to Pseudomonas syringae
Source: PLoS One. 2017 Feb 2;12(2):e0171040. doi: 10.1371/journal.pone.0171040 (PMC5289510; doi:10.1371/journal.pone.0171040)
Supplement: S2 Table — (DOCX) [file pone.0171040.s002.docx]

**S2 Table. Primers Used in This Study**

| ***Primer name*** | ***Sequence(5'-3')*** | ***Application^1^*** |
| --- | --- | --- |
| *Pst* oprf gene-F | AACTGAAAAACACCTTGGGC | (q) |
| *Pst* oprf gene-R | CCTGGGTTGTTGAAGTGGTA | (q) |
| AtRuBisCo-F | GCAAGTGTTGGGTTCAAAGCTGGTG | (q) |
| AtRuBisCo-R | CCAGGTTGAGGAGTTACTCGGAATGCTG | (q) |
| Oligo(dT) | TTTTTTTTTTTTTTTTTTTTTTTTT | Poly-T |
| Actin2-F | CCCGCTATGTATGTCGC | (Q) |
| Actin2-R | AAGGTCAAGACGGAGGAT | (Q) |
| PR1-F | AGGCACGAGGAGCGGTAGG | (Q) |
| PR1-R | CATGTTCACGGCGGAGACG | (Q) |
| PDF1.2-F | TTGCTGCTTTCGACGCA | (Q) |
| PDF1.2-R | TGTCCCACTTGGCTTCTCG | (Q) |

^1^The type of experiment for which the primers were used is indicated in brackets (Q: qRT-PCR, q: qPCR).
